# Characterisation of blood microbiota in patients with liver cirrhosis

**DOI:** 10.1097/MEG.0000000000001494

**Published:** 2019-08-06

**Authors:** Mikio Kajihara, Shigeo Koido, Tomoya Kanai, Zensho Ito, Yoshihiro Matsumoto, Kazuki Takakura, Masayuki Saruta, Kumiko Kato, Toshitaka Odamaki, Jin-zhong Xiao, Nobuhiro Sato, Toshifumi Ohkusa

**Affiliations:** a Department of Gastroenterology and Hepatology, The Jikei University Kashiwa Hospital, Kashiwa; b Division of Gastroenterology and Hepatology, Department of Internal Medicine, The Jikei University School of Medicine; c Next Generation Science Institute, Morinaga Milk Industry Co., Ltd.; d Department of Microbiota Research, Juntendo University Graduate School of Medicine, Tokyo, Japan

**Keywords:** bacterial translocation, liver cirrhosis, microbiota

## Abstract

Bacterial infections arising in patients with liver cirrhosis are associated with life-threatening complications such as hepatic encephalopathy and spontaneous bacterial peritonitis in relation to bacterial translocation. To investigate the state of bacterial translocation, we surveyed the peripheral blood microbiota by *16S rRNA* gene sequencing and analysed the blood microbial profiles.

**Methods:**

Sixty-six patients with liver cirrhosis were enrolled in this study, whereas 14 healthy individuals were also analysed as controls. Total RNA was purified from the peripheral blood, and an approximately 430 base pair genomic locus which included the V3-V4 region of the *16s rRNA* gene was amplified and assessed using bacterial pyrosequencing.

**Results:**

At the genus level, a total of 183 operational taxonomic units were identified in cirrhotic patients, whereas 123 units in controls. Intergroup differences in gut microbiota were analysed by the linear discriminant analysis effect size, which showed that the abundance of *Enterobacteriaceae* was significantly higher in cirrhotics. On the contrary, the abundance of *Akkermansia, Rikenellaceae* and *Erysipelotrichales* were significantly lower in cirrhotics (relative abundance of *Akkermansia* in cirrhotics and controls: 0% and 0.2%, respectively; *Rikenellaceae*: 0.1% and 0.92%; *Erysipelotrichales*: 0.58% and 1.21%).

**Conclusion:**

The present study has demonstrated that various circulating bacteria are present in cirrhotic patients, and the diversity of such bacteria is consistent with the presence of dysbiosis in cirrhotics. Although the clinical significance of the findings remains to be clarified, the findings may potentially facilitate diagnostic and therapeutic attempts to comprehend and furthermore to manipulate bacterial infections in cirrhotic patients.

## Abstract 2

Supplemental Digital Content is available in the text.

## Introduction

Liver cirrhosis (LC) is the common end result of the fibrogenesis that occurs with various chronic hepatic diseases, characterised by the presence of regenerative nodules that cause portal hypertension. The human gut hosts a diverse community of bacteria, and the close relationship between the complications frequently arising in patients with LC and the intestinal microbiota has recently moved into the limelight [1]. The liver is constantly challenged with commensal bacteria and their products arriving via the portal vein in the ‘gut-liver axis’. The mechanism by which bacteria and their metabolites from the intestinal lumen migrate through the intestinal barrier and to mesenteric lymph nodes and, from there, the systemic circulation, is defined as bacterial translocation (BT) [1]. Bacterial infections arising in patients with LC are associated with reduced survival [2], with life-threatening complications such as hepatic encephalopathy (HE) and spontaneous bacterial peritonitis (SBP) caused or aggravated by BT [3]; therefore, preventing bacterial infections in cirrhotic patients is very relevant. However, the ascitic fluid and blood cultures obtained from cirrhotic patients are often negative, even when obtained in optimal conditions [4], probably due to the process of bacterial opsonisation; hence, any subsequent antibiotic therapy remains compelled to be largely empiric.

To further investigate the state of BT, we comprehensively surveyed the peripheral blood microbiota by *16S rRNA* gene sequencing and compared the blood microbial profiles between cirrhotic patients and control subjects.

## Materials and methods

### Patients and controls

Sixty-six patients with LC (44 males and 22 females), who were followed at the Jikei University Kashiwa Hospital, were enrolled in this study. LC was diagnosed on clinical history, physical examination, laboratory tests and imaging studies with or without liver biopsy [5]. The aetiology for LC was: hepatitis B virus (HBV) in seven; hepatitis C virus (HCV) in 36; excessive alcoholic intake [alcoholic liver disease (ALD)] in eight; non-alcoholic steatohepatitis (NASH) in 10 and autoimmune hepatitis or primary biliary cholangitis [autoimmune liver diseases (AILD)] in five. Demographic and clinical findings recorded included age, sex, Child’s grade and the presence or absence of hepatocellular carcinoma (HCC). LC Patients with overt bacterial infections and were on antibiotic therapy at the time of phlebotomy were excluded, and all participants had been antibiotic-free for at least a month before the study. Fourteen unmatched healthy individuals (age: 37.4 ± 10.0 years; sex: 12 males and one female) were also analysed as controls.

The study protocol conformed to the ethical principles of the World Medical Association Declaration of Helsinki as reflected in approval from the Jikei University Institutional Review Board under study number 22-091-9268. All study participants provided written informed consent for the use of their samples in research.

### Sample preparation

Peripheral blood samples were obtained from all subjects. Here, 2.5 ml of whole blood from each subject was collected directly into the PAXgene Blood RNA Tube (PreAnalytiX GmbH, Hombrechtikon, Switzerland) and then cryopreserved until RNA purification. Total RNA was purified by the RNAeasy Mini Kit (Qiagen N.V., Venlo, the Netherlands) and then cDNA was synthesised using the QuantiTect Reverse Transcription Kit (Qiagen N.V.), according to the manufacturer’s instructions, followed by bacterial pyrosequencing. Briefly, 16s bacterial primers Tru357F (5′-CGCTCTTCCGATCTCTGTACGGRAG GCAGCAG-3′) and Tru806R (5′-CGCTCTTCCGATCTG ACGGACTACHVGGGTWTCTAAT-3′) were set to amplify an approximately 430 base pair genomic locus which included the V3-V4 region of the *16s rRNA* gene using the Ex Taq Hot Start Version (TaKaRa Bio Inc., Kusatsu, Japan), as described elsewhere [6].

### Sequence and sequencing data analysis

Pooled amplicon libraries were sequenced employing an Illumina MiSeq platform (2 × 300 bp) and processed further as described in the online supplementary methods section (Supplemental digital content 1, *http://links.lww.com/EJGH/A449*). The QIIME data analysis package version 1.8.0 (http://qiime.org/) was used for 16s rRNA data analysis [7,8]. After removing potential chimeric sequences using UCHIME algorism (http://drive5.com/) [9], sequences were assigned to operational taxonomic units (OTUs) using open-reference OUT picking with a similarity threshold of 97%, then classified taxonomically using the Greengenes reference database (http://greengenes.secondgenome.com/downloads/database/13_5) [10]. Weighed UniFrac distances between subjects and four alpha diversity scores [phylogenetic diversity (PD) whole tree, Chao1, the number of observed species and the Shannon index] were estimated using QIIME version 1.8.0 software.

### Statistical analyses

Each continuous and discontinuous result was expressed as well as the mean ± SD and median (interquartile range), respectively, unless otherwise stated; a *P*-value less than 0.05 was considered significant. To assess the clinical characteristics among cirrhotic patients with different aetiologies, the χ^2^ test was used to compare the frequency of categorical variables and the Kruskal–Wallis test was used to compare the continuous variables.

Intergroup differences of gut microbiota were analysed by the linear discriminant analysis (LDA) effect size (LEfSe) method [11] with default settings on the website (https://huttenhower.sph.harvard.edu/galaxy/root).

## Results

### Demographic and clinical characteristics of patients with liver cirrhosis

Table 1 shows the demographic and clinical characteristics of patients with LC according to their aetiologies. The mean age at examinations was 70.2 years, with no differences between the HBV, HCV, ALD, NASH and AILD subgroups. The LC patients with HBV or ALD were predominantly men, whereas men and women were nearly equally distributed among the HCV-positive patients; however, the difference was not statistically significant. Although the severity of LC, as defined by Child’s grade, was not different, the presence or absence of viable HCC was significantly different between the subgroups (*P* = 0.03).

**Table 1.**
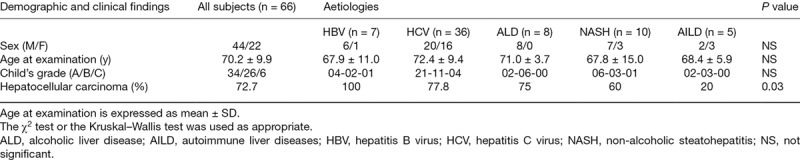
Demographic and clinical characteristics of liver cirrhosis patients according to the different aetiologies

### Detected bacteria in the peripheral blood samples from liver cirrhosis patients

**Fig. 1.**
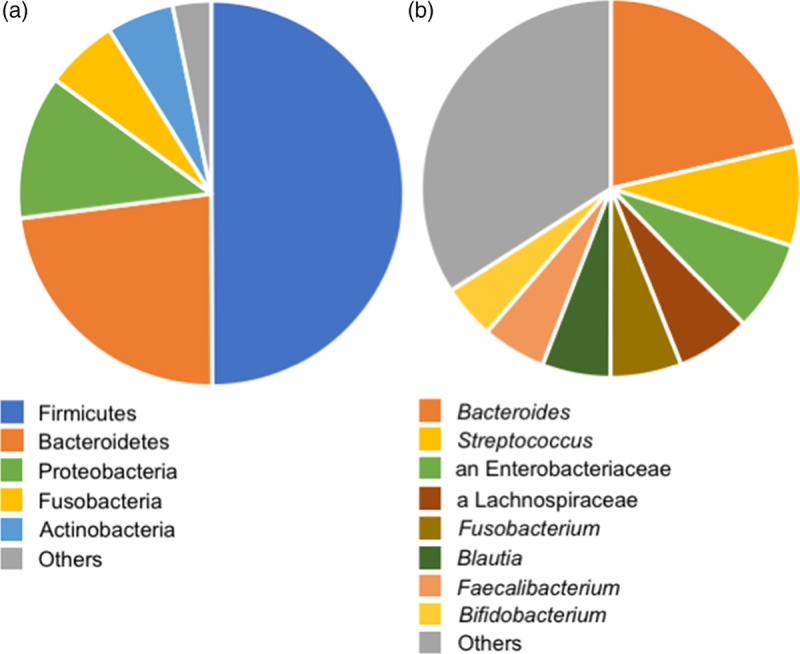
Relative abundances of bacteria at the (a) phylum and (b) genus levels in the peripheral blood samples from LC patients. While five kinds of phyla were dominant at the phylum level (a), the most abundant genus found in LC patients was *Bacteroides*, followed by *Streptococcus*, a genus in the family *Enterobacteriaceae* and a bacteria of the family *Lachnospiraceae* (b). LC, liver cirrhosis.

The relative abundance of microbiota in the peripheral blood samples from LC patients revealed a dominance of five phyla (Fig. 1): Firmicutes (49.9%), Bacteroidetes (23.0%), Proteobacteria (12.1%), Fusobacteria (6.1%) and Actinobacteria (5.6%) (Fig. 1a), but it was hardly different, visually or statistically, from healthy controls. Meanwhile, the most abundant genus found in the LC patients was *Bacteroides* (21.4%), followed by *Streptococcus* (8.5%), a genus in the family *Enterobacteriaceae* (7.8%) and a bacteria of the family *Lachnospiraceae* (6.3%) (Fig. 1b). At the genus level, a total of 183 OTUs were identified in cirrhotic patients, whereas 123 different bacterial genera were distinguished in healthy controls (Supplementary Table S1, Supplemental digital content 1, *http://links.lww.com/EJGH/A449*).

### Microbiota comparisons between groups

**Fig. 2.**
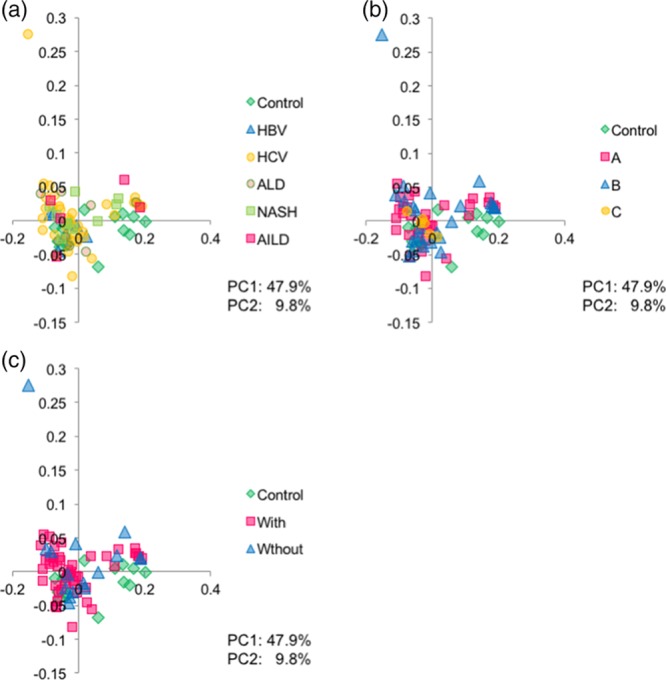
Unweighted UniFrac PCoA of microbiota in the peripheral blood samples from LC patients and healthy controls. (a) Groups defined by aetiology. Control, healthy controls; ALD, alcoholic liver disease; AILD, autoimmune liver diseases; HBV, hepatitis B virus; HCV, hepatitis C virus LC, liver cirrhosis; NASH, non-alcoholic steatohepatitis. (b) Groups defined by severity of LC according to Child-Pugh classification. (c) Groups defined by the presence or absence of viable hepatocellular carcinoma (HCC).

**Fig. 3.**
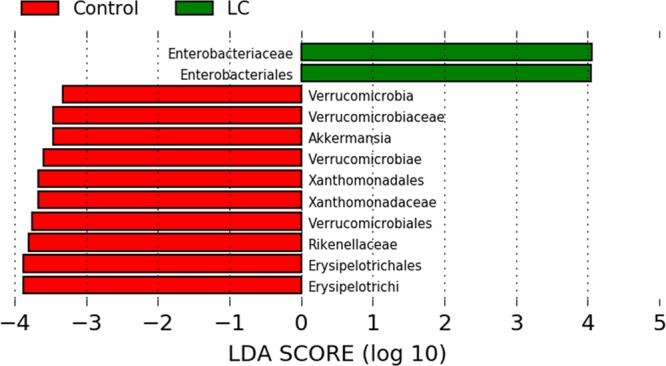
LEfSe results on microbiota in the peripheral blood samples from LC patients and healthy controls. Histogram of the LDA scores computed for features differentially abundant between two groups. LC, liver cirrhosis; LDA, linear discriminant analysis; LefSe, linear discriminant analysis effect size.

**Fig. 4.**
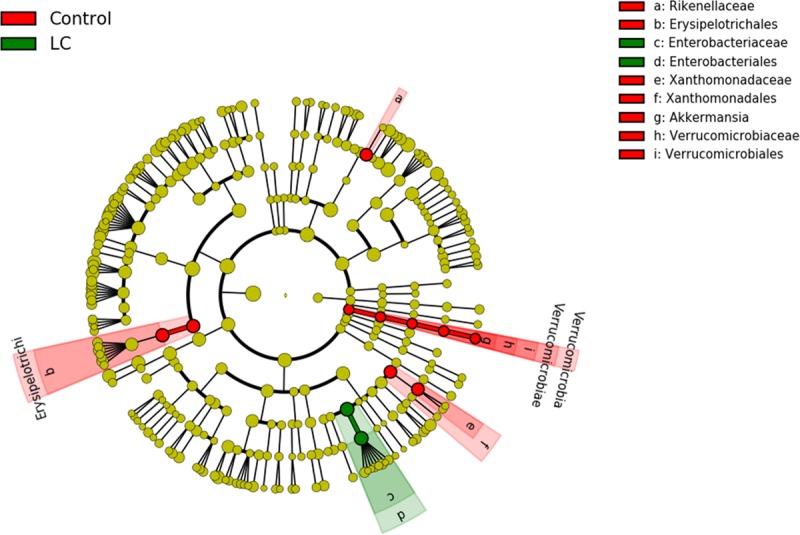
Taxonomic representation of statistically significant differences between two groups. Differences are represented in the colour of the most abundant class. Each circle’s diameter is proportional to the taxon’s abundance.

**Fig. 5.**
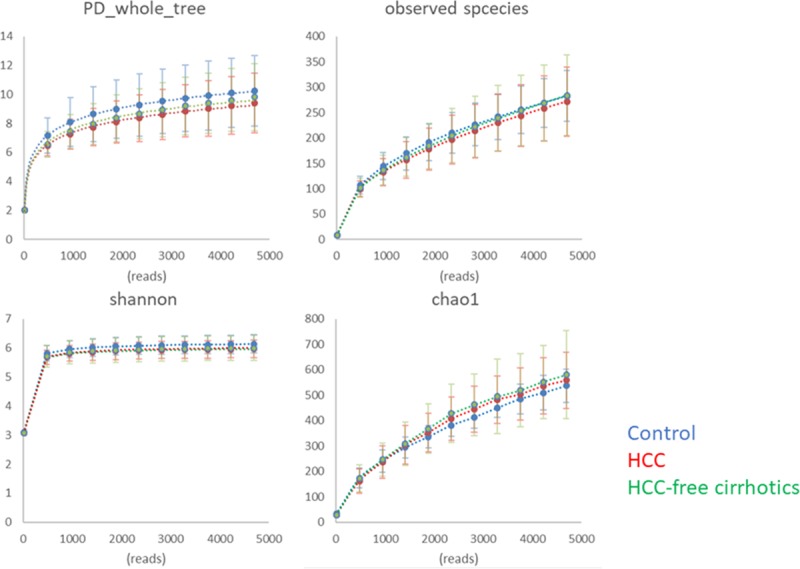
Alpha-diversities of microbiota in the peripheral blood samples from healthy controls (blue), HCC (red) and HCC-free cirrhosis (green). HCC, hepatocellular carcinoma.

Two-dimensional principal coordinates analysis plots of unweighted UniFrac distances were used, based on the relative abundance of OTUs, to visualise complex relationships in the microbial communities between subgroups. There was no obvious separation between groups, defined neither by aetiology (Fig. 2a), by severity (Fig. 2b) of LC, nor presence or absence of viable HCC (Fig. 2c). However, LEfSe analysis showed that the abundance of *Enterobacteriaceae* was significantly higher in cirrhotics (LC patients and healthy controls: 7.8% and 6.0 %, respectively) (Figs. 3 and 4, Supplementary Table S1, Supplemental digital content 1, *http://links.lww.com/EJGH/A449*). On the contrary, the abundance of *Akkermansia*, *Rikenellaceae* and *Erysipelotrichales* was significantly lower in cirrhotics (relative abundance of *Akkermansia* in LC patients and healthy controls: 0% and 0.2%, that of *Rikenellaceae* in LC patients and healthy controls: 0.1% and 0.92%, that of in *Erysipelotrichales* LC patients and healthy controls: 0.58% and 1.21%, respectively) (Figs. 3 and 4, Supplementary Table S1, Supplemental digital content 1, *http://links.lww.com/EJGH/A449*). Likewise, LEfSe analysis showed that LC patients with HCC had the significantly higher abundance of *Enterobacteriaceae* (HCC patients vs. healthy controls: 8.65% vs. 6.02%, respectively) and *Bacteroides* (HCC patients v healthy controls: 23.8% vs. 17.6%, respectively) than healthy controls whilst that of *Bifidobacterium* (HCC patients vs. healthy controls: 5.00% vs. 5.04%, respectively) was significantly lower (Supplementary Table S2, Supplemental digital content 2, *http://links.lww.com/EJGH/A450*). We also calculated the four alpha diversity scores based on PD whole tree, Chao1, the number of observed species and the Shannon index. However, no significant difference was observed between cirrhotic patients and healthy controls (Fig. 5).

## Discussion

The present study has demonstrated that various circulating bacteria are present in patients with LC, based on the positive taxonomy in their peripheral blood samples. Our present findings support the common pathogenic mechanism defined as BT, which explains the passage of bacteria or their products from the intestinal lumen through the intestinal barrier and to mesenteric lymph nodes and eventually to the circulation [1]. Surprisingly, it was not only LC patients but also healthy controls, who proved positive for microbiota in peripheral blood samples, indicating that even healthy subjects are constantly challenged with commensal bacteria and meaning that BT is a universal phenomenon, not exclusive to cirrhosis. Even so, it is the cirrhotics in which BT has the significance in the clinical settings while little or no clinical significance in healthy subjects, and it is probably because BT is peculiarly facilitated in LC, in which intestinal bacterial overgrowth, impairment in intestinal permeability and most significantly deficiencies in local host immune defences are co-existent [12]. Actually, the symptomatic infections in patients with cirrhosis are common, with the incidence being four to five times greater than in the general population [13]; our findings are also in line with this.

It is widely recognised that infections contribute to a high morbidity and mortality in cirrhotic patients. Once infection occurs, patients with LC are vulnerable to further infections and complications, that is, acute kidney injury, de-listing from liver transplants and prolonged hospital stays [13]. Bacterial infections arising in patients with LC are also associated with significantly reduced survival, with a four-fold increase in mortality for cirrhotics with infection compared with similar LC patients without infection [2], because these infections are considered to be associated with life-threatening complications such as HE and SBP [3]. Therefore, preventing bacterial infections in cirrhotic patients is very relevant.

The diagnosis of infections in cirrhosis, however, could be challenging. Ascitic fluid and blood cultures obtained from cirrhotic patients are often negative, even when obtained under optimal conditions [4]. Actually, using conventional culture techniques, ascitic fluid cultures could be negative in up to 60% of patients with clinical manifestations suggestive of SBP, despite the use of sensitive methods [14]. The reason for this remains undetermined, but it is probably due to the relatively low concentration of bacteria in specimen as well as the process of bacterial opsonisation. Despite negative ascitic and blood cultures, patients with suspected infection, such as increased ascites polymorphonuclear leucocytes count, should be considered as having one, but as a consequence of the frequently negative cultures, the subsequent antibiotic therapy remains compelled to be largely empiric. With respect to SBP, the Gram-negative aerobic bacteria from the family of *Enterobacteriaceae* and non-enterococcal *Streptococcus* spp. are conceived to be the most common causative organisms [14]; the initial empiric antibiotic therapy is required to cover them. In the present study, the second and third most abundant genera found in the cirrhotic patients were *Streptococcus* and a genus in the family *Enterobacteriaceae*, respectively, and this could be compatible with the previous concepts based on conventional fluid cultures and warrant the conventional use of such empiric antibiotics as cefotaxime.

In the meantime, there can be an argument that these bacteria are of little pathological significance, because the microbiota was anyhow detected in all of their samples, although none of the LC patients enrolled in the study had concomitant overt bacterial infection or were in need to be on systemic antibiotics at the time of study. The current study is also limited because blood microbiome characteristics were not found to be associated with clinical severity of cirrhosis, in contrast with previous studies on gut microbiome analysing stool samples [15,16]. Regarding this point, further studies of larger sample sizes, enrolling LC patients with or without overt bacterial infections, as well as healthy cohort matched for age and sex distribution, and moreover, by the use of both blood and stool samples, should be required next to verify the pathogenic significance of circulating microbiota. Even so, blood microbial communities are distinct in patients with LC compared with healthy controls, and the cirrhotic patients of diverse aetiologies shared similar microbial profiles, analysed using UniFrac studies, which could lead to the conclusion that the altered microbial profiles in LC patients are likely to result from cirrhosis development irrespective of the aetiologies, and not *vice versa*, and hence our new findings are of interest nonetheless.

The pathological significance of the prevalence of the likes of family *Enterobacteriaceae* in patients with HCC also remains to be clarified. This could just be a consequence of carcinogenesis, but several lines of evidence, both experimental and clinical, suggest that the intestinal microflora is critically involved in the pathogenesis of HCC by creating a lipopolysaccharide-dependent pro-inflammatory microenvironment of the liver [17]. In particular, in a mouse model of obesity-induced HCC, it was demonstrated that deoxycholic acid produced by gut microbiota could potentiate tumour development by provoking the senescence-associated secretory phenotype in hepatic stellate cells and upregulation of interleukin 6 [18]. Therefore, the present findings seem to be of great interest.

In conclusion, this study delivers one of the first applications of *16S rRNA* gene sequencing to characterise the microbial profiles of peripheral blood of LC patients in comparison with healthy controls and the findings, showing that the blood microbial communities are different in patients with LC from healthy individuals. Although the clinical significance of the distinction, that is, whether the change in microbial compositions is cause or result of cirrhosis development, remains to be elucidated, because of the relative accessibility of peripheral blood compared with faeces, the findings will potentially facilitate diagnostic attempts to comprehend the actual state of BT with its resulting complications such as HE and SBP, and to manipulate intestinal microbiota for cirrhotic patients through the optimised administration of probiotics and antibiotics.

Further studies are eagerly anticipated to characterise the functions and pathological significance of peripheral blood bacteria in LC.

## Acknowledgements

We would like to thank Mr. Sankichi Horiuchi for his support and assistance in the sample processing.

This work was performed primarily within the internal research budgets of the authors institutions.

Patient consent: Obtained.

The study was approved by the Jikei University Institutional Review Board under study number 22-091-9268.

M.K., S.K., T.K., Z.I., Y.M., K.T. and T. Odamaki designed and executed the clinical research study. M.K., T. Odamaki and T. Ohkusa were the principal investigators. K.K., T. Odamaki and J.-z.X. analysed the data and performed statistics. M.S. and N.S. supported data integration and project management. M.K. wrote the manuscript. All authors reviewed and approved the manuscript.

## Conflicts of interest

K.K., T. Odamaki and J.-z.X. are employed by Morinaga Milk Industry Co., Ltd., which manufacture probiotic products marketed worldwide. For the remaining authors, there are no conflicts of interests.

## Supplementary Material
